# Prostaglandins limit nuclear actin rod formation during *Drosophila* oogenesis

**DOI:** 10.17912/micropub.biology.001571

**Published:** 2025-04-03

**Authors:** Tina L. Tootle

**Affiliations:** 1 Biology, University of Iowa, Iowa City, Iowa, United States; 2 Anatomy and Cell Biology, University of Iowa Carver College of Medicine, Iowa City, Iowa, United States

## Abstract

Expression of GFP-Actin results in nuclear actin rod formation during specific stages of *Drosophila melanogaster* oogenesis. Loss of prostaglandin (PG) synthesis and signaling results in an increased frequency of cells with nuclear actin rods; there are less rods per cell, but the rods are longer. These findings suggest that loss of PGs results in increased nuclear actin and are consistent with prior findings assessing the roles of PGs in modulating endogenous nuclear actin. Thus, GFP-Actin rod formation can be used as a tool to screen for new regulators of nuclear actin.

**Figure 1. Prostaglandins limit nuclear GFP-Actin rod formation:**
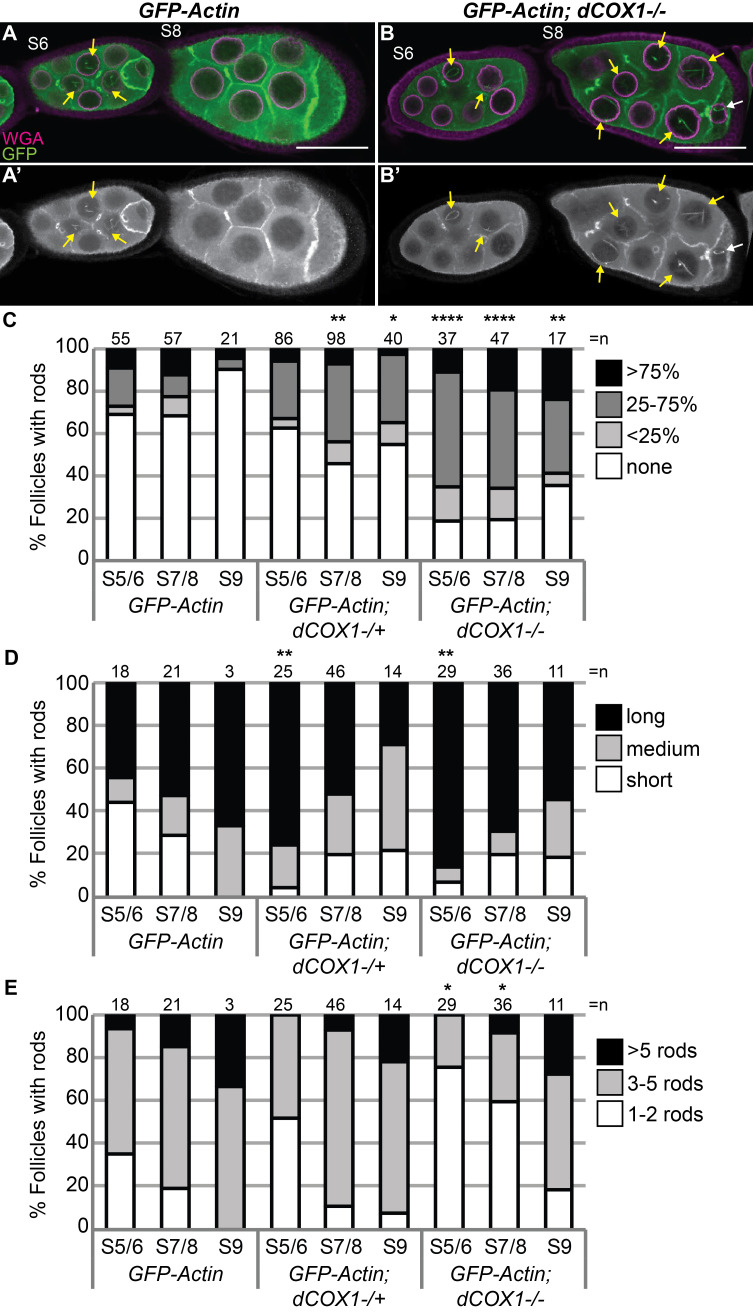
A-B’. Maximum projections of 2-3 confocal slices of *osk GAL4/UAS GFP-Actin 5C* ( *GFP-Actin* ) and *osk GAL4/UAS GFP-Actin 5C* ; *dCOX1-/-* ( *GFP-Actin; dCOX1-/-* ) follicles of the indicated stages (S) stained for: nuclear envelope (WGA, magenta in merge) and anti-GFP (green in merge). Yellow arrows indicate nuclear actin rods in the nurse cells and white arrows indicate those in the oocyte. Scale bars = 50μm. C-E. Graphs quantifying the percentage of follicles of the indicate stages and genotypes exhibiting the indicated phenotypes; ****p<0.0001, **p<0.01, *p<0.05 (Fisher’s exact test). n = number of follicles. The graph in C shows the frequency of follicles with no (white), <25% (light gray), 25-75% (dark gray), and >75% (black) of nurse cells with GFP-Actin rods. For the follicles exhibiting rods the length of the rods (D) and the number of rods (E) were quantified. Reduction or loss of dCOX1 results in an increased frequency of nurse cells with nuclear GFP-Actin rods (A-C), and in the early stages these rods are longer (D). Further, loss of dCOX1 results in a reduced number of nuclear GFP-Actin rods per nuclei (E).

## Description

Actin localizes and functions within the nucleus. The import and export of actin from the nucleus is highly regulated (Borkuti et al., 2022; Dopie et al., 2012; Stuven et al., 2003), supporting that it has important nuclear functions. Indeed, recent work reveals nuclear actin has a wide variety of activities, including regulating transcription, being a component of chromatin remodeling complexes, mediating DNA damage repair, and contributing to nuclear structure (Green et al., 2021; Hurst et al., 2019; Kelpsch & Tootle, 2018; Misu et al., 2017). The functions of nuclear actin, like cytoplasmic actin, are regulated by its form – monomeric, polymeric, filamentous, and networks of filaments (Fukui & Katsumaru, 1979; Grosse & Vartiainen, 2013; Hendzel, 2014; Kristo et al., 2016; Vartiainen, 2008). In some cases, thick nuclear actin filaments are observed; these are often referred to as nuclear actin rods. Such rods form when there is a large influx of actin into the nucleus; this occurs in the contexts of cellular stress (Fukui & Katsumaru, 1979; Iida et al., 1986; Munsie et al., 2012; Nishida et al., 1987; Osborn & Weber, 1980; Vartiainen et al., 2007). Thus, nuclear actin rods are indicative of high nuclear actin levels. While nuclear actin plays critically important roles in cellular functions, the mechanisms regulating its level and form remain poorly understood.

We established *Drosophila* oogenesis as a new, in vivo system for studying nuclear actin regulation and function. Female flies have two ovaries, each of which contains 15-20 ovarioles or chains of sequentially developing egg chambers or follicles. There are fourteen stages of follicle development (Giedt & Tootle, 2023). Each follicle is comprised of one oocyte, fifteen germline-derived nurse cells and a layer of epithelial cells termed follicle cells. We previously found that during follicle development there are multiple pools of endogenous nuclear actin that exhibit unique developmental patterns (Kelpsch et al., 2016; Wineland et al., 2018). Further, germline expression of exogenous actin labeling tools (Lifeact-GFP, Utrophin-GFP, and GFP-Actin) perturbs nuclear actin dynamics and results in stage-dependent nuclear actin rod formation (Kelpsch et al., 2016; Spracklen, Fagan, et al., 2014). These rods form due to the tools artificially stabilizing endogenous nuclear actin (Lifeact-GFP or Utrophin-GFP) and/or increasing nuclear actin levels (GFP-Actin).

Using *Drosophila* oogenesis as a model, we identified prostaglandin (PGs) signaling as a novel regulator of nuclear actin. PGs are locally acting lipid signaling molecules that are produced in a multi-step process (Funk, 2001; Tootle, 2013). Briefly, the fatty acid arachidonic acid, the substrate for PG production, is enzymatically released from lipid stores. This substrate is acted on by cyclooxygenase (COX) enzymes and PG-type specific synthases to produce bioactive PGs; these PGs then activate G protein-coupled receptors to mediate their downstream functions. Flies have a single COX-like enzyme termed Pxt/dCOX1, subsequently referred to as dCOX1 (Tootle & Spradling, 2008). We previously found that during *Drosophila* oogenesis PGs regulate both actin cytoskeletal remodeling and nuclear actin (Groen et al., 2012; Spracklen, Kelpsch, et al., 2014; Talbot et al., 2023; Tootle & Spradling, 2008). Specifically, PG synthesis and signaling normally limits the amount and regulates the forms of nuclear actin (Talbot et al., 2023).

To further test the role of PG signaling in controlling nuclear actin, we asked whether PGs regulate nuclear actin rod formation. Germline expression of GFP-Actin increases nuclear actin levels, and this, in turn, induces nuclear actin rod formation in a low frequency of nurse cells during Stages 5-9 (S5-9) (Kelpsch et al., 2016). As loss of dCOX1 increases endogenous nuclear actin (Talbot et al., 2023), we hypothesized that *dCOX1* mutant follicles will exhibit increased GFP-Actin rod formation. Indeed, that is what we observe (Fig 1B-B’ compared to A-A’). To quantify this change follicles were binned into 4 categories based on the percentage of nurse cells exhibiting actin rods – 0, <25%, 25-75%, and >75%. Both reduction in (heterozygosity) or loss of dCOX1 increases the frequency of follicles exhibiting actin rods (Fig 1C). We also assessed rod length (short, medium, or long), and number of rods per nuclei. Reduction or loss of dCOX1 results in longer rods during S5/6 (Fig 1D), and in *dCOX1* mutants the number rods are fewer during S5/6 and S7/8 (Fig 1E). These data are consistent with the idea that small rods are joined to create larger and fewer rods as nuclear actin levels increase (Ishikawa-Ankerhold et al., 2017); however additional experiments are needed to formally test this possibility. The increased GFP-Actin rod formation observed when dCOX1 is lost correlates with our prior finding that PG synthesis and signaling are required to limit the level of endogenous nuclear actin (Talbot et al., 2023). Therefore, alterations in GFP-nuclear actin rod formation can be used to screen for regulators of nuclear actin.

## Methods

**Fly stocks**

Fly stocks were maintained on cornmeal-agar-yeast food at 21°C. The following stocks were obtained from the Bloomington Drosophila Stock Center (Bloomington, IN): *yw* (RRID: BDSC_1495) and UASp GFP-Actin5C (RRID: BDSC_9258). *pxt ^f01000^* (referred to as *dCOX1-/-* ) was obtained from the Harvard Exelixis collection (Boston, MA). The *oskar GAL4* line was a generous gift from Anne Ephrussi (Telley et al., 2012); this stock can also be obtained from the Bloomington Drosophila Stock Center (RRID: BDSC_44241). Expression of UASp GFP-Actin 5C was achieved by crossing to *oskar GAL4* flies, maintaining fly crosses at 21°C, and maintaining progeny at 25°C for 5-6 days, during which the flies were fed wet yeast paste daily.

**Immunofluorescence**

Whole-mount *Drosophila* ovary samples were dissected into room temperature Grace’s insect medium (Lonza, Walkersville, MD). Samples were fixed for 10 min at room temperature in 4% paraformaldehyde in Grace’s insect medium. Samples were blocked by washing in antibody wash (1X phosphate-buffered saline, 0.1% Triton X-100, and 0.1% bovine serum albumin) six times for 10 mins each at room temperature. The primary antibody was incubated overnight at 4°C; rabbit anti-GFP 1:2000 (pre-absorbed on *yw* ovaries at 1:20 and used at 1:100; RRID: AB_10013661, Torrey Pines Biolabs, Inc., Secaucus, NJ). After 6 washes in antibody wash (10 min each), secondary antibodies were incubated overnight at 4°C or for ~4 hours at room temperature. The following secondary antibody was used at 1:250-1:500: AlexaFluor 488:goat anti-rabbit (RRID:AB_2576217). AlexaFluor 555-conjugated wheat germ agglutinin, WGA (1:500; ThermoFisher Invitrogen) was included with the primary and secondary antibodies. Following six washes in antibody wash (10 min each), 4′,6-diamidino-2-phenylindole (DAPI, 5 mg/ml) staining was performed at a concentration of 1:5000 in 1X PBS for 10 min at room temperature. Samples were then rinsed in 1X PBS and mounted in 1 mg/mL phenylenediamine in 50% glycerol, pH 9 (Platt & Michael, 1983). All experiments were performed a minimum of three independent times.

**Image acquisition and processing**

Microscope images of fixed *Drosophila* follicles were obtained using Zen software on a Zeiss 700 mounted on an Axio Observer.Z1 using a Plan-Aprochromat 20x/0.8 WD=.55 M27 (Carl Zeiss Microscopy, Thornwood, NY). Maximum projections, merged images, rotation, and cropping were performed using ImageJ software. All fluorescent images were brightened by 30% in Photoshop (Adobe) to improve visualization.

**Quantification of nuclear actin rod frequency, length, and number**

Quantification of nuclear actin rods was performed on confocal image stacks of follicles stained with anti-GFP, WGA, and Phalloidin. Genotypically de-identified images were analyzed using ImageJ; as necessary, brightness and contrast were adjusted to score all the actin rods present. Data was collected for S5-6, S7-8, and S9; follicle staging was assigned based on morphology and size. For each follicle the percentage of nurse cells exhibiting nuclear actin rods was assessed and binned into four categories: none, ≤25%, 25-75%, or ≥75%. For each follicle with nurse cells exhibiting nuclear actin rods, the number of rods was counted and binned into 3 categories: 1 to 2 rods, 3 to 5 rods, or >5 rods per nuclei. The rods were then scored for length: short (≤1/4 diameter of nucleus), medium (~1/2 diameter of nucleus), or long (≥1 diameter). Data was analyzed using Excel (Microsoft) and statistical analysis was preformed using R (Vienna, Austria).

## Reagents

**Table:** 

**Organisms/Strains**			
***Drosophila* Strains **	**Genotype**	**Identifier**	**Available from**
*yw*	y ^1^ w ^1^	RRID: BDSC_1495	Bloomington Drosophila Stock Center (BDSC)
*oskar* GAL4	w ^1118^ ; P{osk-GAL4::VP16}A11/CyO	FBtp0083699 Also: RRID: BDSC_44241	from A. Ephrussi Also available from: BDSC
UAS- *GFP-Actin 5C*	w[*]; P{w[+mC]=UASp-GFP.Act5C}2-1	RRID: BDSC_9258	BDSC
* pxt ^f01000^* (referred to as *dCOX1-/-* )	PBac{WH}pxt ^f01000^	FBal0178293	Harvard Exelixis Collection; Thibault, 2004 PMID: 14981521

**Antibodies**			
**Name**		**Identifier**	**Source**
rabbit polyclonal anti-GFP (TP401)		RRID: AB_10013661	Torry Pines Biolabs, Inc

**Reagents**			
**Name**		**Identifier**	**Source**
AlexaFluor 555 Wheat Germ Agglutinin		Cat# W32464	ThermoFisher Invitrogen
4’,6-Diamidino-2-phenylindole (DAPI)		Cat# D9542	Millipore Sigma
bovine serum albumin		Cat# A7284	Sigma-Aldrich
Grace's Insect Media		Cat# 04-457F	Lonza
